# Neuromodulation of the Dorsal Root Ganglion for Chronic Postsurgical Pain

**DOI:** 10.1093/pm/pnz072

**Published:** 2019-06-01

**Authors:** Ajay B Antony, B Carsten Schultheis, Suneil M Jolly, Daniel Bates, Corey W Hunter, Robert M Levy

**Affiliations:** 1 University of Florida College of Medicine, Gainesville, Florida, USA; 2 Krankenhaus Neuwerk “Maria von den Aposteln,” Mönchengladbach, Germany; 3 Louisiana Pain Specialists, New Orleans, Louisiana, USA; 4 Metro Pain Group, Victoria, Australia; 5 Ainsworth Institute of Pain Management, New York, New York; 6 Institute for Neuromodulation, Boca Raton, Florida, USA

**Keywords:** DRG Stimulation, Complex Regional Pain Syndrome, Spinal Cord Stimulation, Postsurgical Chronic Pain

## Abstract

**Objective:**

The objective of this study is to review the available evidence for dorsal root ganglion (DRG) stimulation for the treatment of complex regional pain syndrome type II (CRPS II; peripheral causalgia) associated with chronic neuropathic postsurgical pain (NPP).

**Design:**

Available literature was identified through a search of the US National Library of Medicine’s Medline database, PubMed.gov. References from published articles also were reviewed for relevant citations.

**Results:**

The data published to date support the use of DRG stimulation to treat chronic NPP of the groin, knee, and foot. NPP following procedures such as thoracotomy, hernia surgery, and knee replacement surgery were identified as some of the conditions for which DRG stimulation is likely to be effective.

**Conclusion:**

DRG stimulation is known to be an effective treatment for focal neuropathic pain. Currently, NPP of the foot, groin, and knee all appear to be the conditions with the most clinical experience, backed by a limited but growing body of evidence. However, prospective studies lag behind real-world clinical experience and are needed to confirm these findings.

## Introduction

Chronic postsurgical pain is an important cause of morbidity that is often neuropathic in nature and has historically been difficult to treat [1,2]. Stimulation of the dorsal columns, while effective for treating broad, nonspecific regions of pain, has had well-documented shortcomings in treating focal neuropathic pain—the hallmark of neuropathic postsurgical pain (NPP) [3,4]. Fortunately, a new and innovative neuromodulation therapy is available that allows stimulation to be focused on specific painful areas/regions of the body, thus addressing one of the limitations of dorsal column spinal cord stimulation (SCS) in conditions like NPP. In 2016, Deer et al. published the results of the ACCURATE study (a prospective, multicenter randomized clinical trial) that showed dorsal root ganglion (DRG) stimulation to be superior to conventional dorsal column stimulation for the treatment of complex regional syndrome (CRPS) type I (reflex sympathetic dystrophy [RSD]) and type II (causalgia) [5]. In the ACCURATE study, a diagnosis of peripheral causalgia or CRPS type II was made in accordance with accepted diagnostic criteria, that is, pain resulting from known damage to a “named” nerve (i.e., saphenous nerve, ilioingional nerve, etc.) “in an anatomical area consistent with the innervation pattern of the damaged nerve (or nerves), and generally, in a hyperalgesic state” [6]. In addition to significant improvements in pain and quality of life, the ACCURATE study DRG cohort also reported significantly less paresthesia, minimal postural variation, and reduced paresthesia outside of the targeted painful area as compared with the control group (dorsal column spinal cord stimulation) [5].

DRG stimulation was approved for use in Europe in 2011 and Australia in 2012. In Europe and Australia, DRG stimulation is indicated for treatment of patients with chronic, intractable pain. DRG stimulation was approved for use in the United States by the Food and Drug Administration in 2015 for patients with chronic intractable pain of the lower limbs associated with a diagnosis of CRPS I and CRPS II (peripheral causalgia).

Since then, DRG stimulation has continued to be successful in the treatment of CRPS [7,8] as well as a variety of other focal, postsurgical, neuropathic pain syndromes like post-herniorrhaphy neuralgia, phantom limb pain, and persistent pain after total joint replacement [9–12]. Subsequent publications examining DRG stimulation have continued to replicate the success reported in the ACCURATE study, thus showing the durability and reproducibility of this therapy [9,13]. Liem et al. reported significant improvements in mood, pain, and quality of life with DRG stimulation therapy through 12 months of therapy for a variety of neuropathic conditions [13]. Most recently, Hunter et al. presented the results of a multicenter study of 217 patients treated with DRG stimulation, 44 of whom had postsurgical pain syndromes. The authors reported statistically significant reductions in pain across all diagnoses, with a trial:permanent ratio similar to that reported in the ACCURATE study [9].

This article will present a review of DRG stimulation for the treatment of peripheral causalgia (CRPS II) associated with chronic postsurgical pain due to nerve injury.

## Methods/Results

### Groin/Hernia Surgery (Post-herniorrhaphy Neuralgia)

More than 20 million people undergo inguinal hernia surgery annually around the world, and it is estimated that up to 16% of these individuals will develop chronic and severe postoperative pain, known as post-herniorrhaphy neuralgia [14–17]. This condition can often be neuropathic in nature, characterized by burning pain or even allodynia in the distribution of the ilioinguinal, iliohypogastric, and/or the genital branch of the genitofemoral nerve. The mechanisms of injury can be the result of direct structural damage as a byproduct of the surgical procedure (i.e., excessive traction or pressure on one of the aforementioned nerves), stitch or mesh entrapment, postoperative inflammation, and/or scar tissue formation [18].

Treatment of neuropathic groin pain traditionally involves a stepwise approach that includes conservative management with oral analgesics and anti-epileptics, interventional pain modalities like nerve blocks and ablations, peripheral nerve stimulation, surgical revision of the repaired groin, or even a surgical neurectomy. Corticosteroid injections and neuroablative techniques (i.e., cryoablation and radiofrequency ablation) have been frequently utilized; however, the relief tends to be short term [19–21].

Surgical neurectomy of the genitofemoral, ilioinguinal, and iliohypogastric nerves (aka “triple neurectomy”) is an accepted and sometimes preferred treatment for surgeons for post-herniorrhaphy neuralgia refractory to conservative management. However, there have been no published studies demonstrating the long-term efficacy of this technique [22]. Although this procedure is widely implemented, the risks of this particular surgery are considerable (i.e., permanent sensory loss, loss of the cremasteric reflex, and differentiation pain) and should not be thought of as a benign intervention, as destruction of peripheral nerves may actually increase ectopic firing and even lead to allodynia [22,23].

Dorsal column stimulation has been used to treat cases of postsurgical groin pain; however, paresthesia coverage has traditionally been difficult to achieve and maintain long term in the groin [3,24]. Even in cases where coverage in the affected groin is accomplished, patients will often complain of unwanted paresthesia in nearby unaffected areas due to the need for high stimulation parameters to achieve paresethesia in the groin in the first place. Compounding matters are the well-documented variations in stimulation intensity due to postural changes associated with tonic dorsal column SCS. This variation in stimulation intensity can cause the perceived paresthesia to become painful and increase the incidence of collateral paresthesia [25]. The DRG is a particularly attractive target for post-herniorrhaphy neuralgia due to the ability to isolate stimulation in the groin, program at subperception settings, and avoid posture-based changes [26].

Post-herniorrhaphy neuralgia is one of the most compelling uses for DRG stimulation due to the excellent pain relief and consistent reproducibility provided by the therapy. In 2015, Schu et al. reported the results of a retrospective study of 29 patients treated with DRG stimulation, including 12 patients with post-herniorrhaphy pain. The average decrease in visual analog scale (VAS) was 71% at seven months in the 25 of 29 patients who went on to receive implanted systems. In the post-herniorrhaphy pain subgroup, 10 of 12 patients had a successful trial, and half of those had >80% reduction in their symptoms [27]. In 2016, Levine at al. published a prospective, single-center observational study of 32 patients, reporting success in treating neuropathic groin pain with DRG stimulation [28]. In the largest prospective study of DRG therapy for chronic neuropathic groin pain, Morgalla et al. reported on 30 patients followed to three months and 11 followed out to three years with significant improvements in groin pain. Secondary outcomes of disability (Pain Disability Index), catastrophizing (Pain Catastrophizing Scale), overall pain severity and functional impact (Brief Pain Inventory), and depression (Beck Depression Inventory) were all reduced, with statistical significance (*P* < 0.05) [29]. In this study, the most common levels of DRG lead placement were L1 and L2, although the authors suggested that T12 may be a useful target as well. The sensory input of the groin can vary from lower thoracic to midlumbar nerve roots; depending on the nature of the pain or nerve injury, branches of the ilioinguinal nerve, genitofemoral nerve, or both could be involved. Transforaminal paresthesia mapping with 50-Hz stimulation has been reported to have success in leading to successful correlation with intraoperative DRG stimulation [30]. Reports of successful treatment of neuropathic groin pain with DRG stimulation continue to emerge [31,32]. Based on the currently published data on DRG stimulation for post-herniorrhaphy neuralgia, the authors are able to give a strong recommendation for its utility here; however, a high-powered prospective trial with a control group is needed to further that support.

### Post–Joint Surgery of the Knee and Hip

DRG stimulation has also been shown to be beneficial for treating cases of postsurgical joint pain in the knee and hip [9,33]. There are >700,000 total knee replacement (TKR) surgeries and revisions each year in the United States, and the incidence of NPP can be as high as 34% [32,34]. Intuitively, DRG stimulation may not seem like an appropriate treatment in this particular subset of patients, as joint pain tends to be viewed as nociceptive. However, there may be a neuropathic element present that would explain the persistence of pain in an artificial joint. In the DRG FOCUS study, the authors reported on 14 patients (12 knee, two hip) with pain after total joint replacements who were successfully treated with DRG stimulation [9].

In 2018, Morgalla et al. reported the outcomes of 27 patients implanted with DRG stimulation for the treatment of postoperative knee pain, 16 of whom were followed up to three years. The average reduction of VAS in this group was 69% [35]. A case report by van Bussel et al. also showed a patient with CRPS of the knee, secondary to a diagnostic arthroscopic procedure, responding favorably to DRG stimulation of L2, L3, and L4. At three-month follow-up, the patient reported that an initial numeric rating scale (NRS) score of 9 had dropped to a 1–2, and the patient stated that the movement of the knee had improved [7]. Postsurgical neuropathic knee pain, regardless of adherence to the diagnostic criteria for CRPS I or II, is effectively treated with DRG stimulation. With the volume of knee surgery increasing and substantial chronic postsurgical pain rates, the body of prospective evidence seems likely to expand.

Total hip arthroplasty is another common orthopedic surgery that exhibits similar postoperative pain trajectories as knee replacement surgery. It is estimated that 950,000 primary and revision THAs were performed globally in 2010 [36]. The majority of patients describe significant improvements in pain and functionality after THA [36]. However, not all benefit from total hip arthroplasty, and the prevalence of persistent postsurgical pain after THA ranges between 7% and 23%. Of this patient group, about one-third present signs of neuropathic pain after THA [37]. A study of DRG stimulation for multiple etiologies reported on two patients with post-THA neuropathic pain who were successfully treated with DRG stimulation [9]. Anecdotal findings exist for the successful use of DRG therapy for postsurgical hip pain, and these data should be explored in further studies. Current evidence for DRG stimulation for post-THA pain is behind that for postsurgical knee pain but seems to be a promising indication for the therapy.

### Amputation

DRG stimulation is a logical option for treatment of phantom limb pain (PLP); however, publication on the topic has been limited to date [10,38,39]. Postamputation patients by definition have a nerve injury (neurotomy), and many suffer chronic stump, residual limb, and/or phantom pain. Nerve injury associated with PLP creates a hyperexcitable state with ectopic neuronal activity of the DRG [20,23]. Direct stimulation of the DRG amplifies low pass filtering at the T junction within the DRG [40]. The underlying pathophysiology combined with an evolving understanding of mechanisms of action suggests DRG stimulation to be an ideal treatment option for PLP. As a CRPS-type syndrome, many of these nerve injury patients demonstrate signs of allodynia, hyperpathia, and temperature changes in the residual limb. Eldabe was first to publish on DRG stimulation in PLP, showing a high trial success rate with all patients proceeding to implant. Long-term follow-up demonstrated an average 52% decrease in pain, with the average VAS decreasing from 83.5 +/− 10.5 to 38.9 +/− 27.1 [10]. Hunter, et al. [41] successfully demonstrated that radiofrequency stimulation can be used to predict the optimal DRG target in a series of four PLP patients, eventually demonstrating 60–90% pain relief after implant. This valuable technique may help clarify the optimal target DRG(s) to stimulate in cases of PLP where the affected or painful dermatomes are surgically absent and neuroplastic changes may create overlap or shifts from expected coverage regions. Most recently, a case of successful DRG stimulation following failed conventional SCS was reported in a patient with recurrent CRPS after amputation [42]. Interestingly, single-level DRG stimulation at L4 gave initial pain relief of 25%, but this increased substantially to 60% over 17 months, enough to allow wearing of a prosthesis. DRG stimulation uniformly has shown a high level of success in treating patients with PLP.

### Pelvic Pain

Chronic pelvic pain (CPP) is a debilitating, highly prevalent, and costly condition that affects up to 25–40% of both men and women all over the world [42]. Although this condition includes a variety of diagnoses such as pudendal neuralgia, interstitial cystitis, and endometriosis, common themes with CPP include a cumbersome and often lengthy diagnostic process with limited therapeutic options [41]. Strong links can be found between CPP and CRPS, the primary indication for DRG stimulation. Diagnostic criteria for CRPS may actually be present in a subset of CPP patients; however, clinical diagnosis is most often not carried out due to the internal nature of the affected tissues [43]. Chronic pelvic pain is also strongly linked to psychological factors such as catastrophizing, fear-avoidance behavior, and emotional disability [44]. Treatment options traditionally have included physical therapy, psychological and behavior modification, medications, and surgery [45]. Surgical options for pelvic floor disorders, specifically implantable mesh therapies, are becoming known risk factors for the development of new CPP [46]. Despite the strong link to CRPS, CPP has the highest rate of explant of all diagnoses for which SCS has been used. The two most common reasons for explant are the inability to achieve or maintain stimulation in the affected pelvic area and the presence of collateral paresthesia in nearby unwanted areas [45].

Spinal cord stimulation for pelvic pain has been studied at the midthoracic level, the conus medularis, and sacral nerve roots [47–49]. Large variations and lack of consensus on anatomical targets have made clinical decision-making difficult, and high explant rates have left neuromodulators searching for alternative options [50]. DRG stimulation for the treatment of CPP may offer effective and consistent neural targeting. The first case study of CPP treated with DRG stimulation described L1 and L2 leads providing sustained relief for pelvic girdle pain [51]. In 2018, Hunter and Yang also reported on the successful use of DRG stimulation for the treatment of CPP in a seven-patient case series; however, the authors described utilization of the L1 and S2 DRG levels [52]. The authors suggested the highly complex innervation of the pelvic region and the involvement of eight different nerves on each side capable of nocioceptive conduction as the reason for the predictably low success rate with SCS for CPP. By targeting the L1 and S2 levels, the authors proposed that they would be able to provide stimulation to at least one spinal segment of six out of the eight pelvic nerves and increase the chances for success (Table 1). This proposed standardization of lead placement shows promise and warrants prospective study in the future, as the authors demonstrated decreases in opioid consumption and pain relief out to one year.

**Table 1:** Major nerves of the pelvic region and associated spinal segments

Nerve	T12	L1	L2	L3	L4	S1	S2	S3	S4	S5
Iliohypogastric										
Ilioinguinal										
Genitofemoral										
Obturator										
Posterior femoral cutaneous										
Inferior rectal										
Pudendal										
Coccygeal										

Gray shading illustrates the extent of coverage by simultaneously stimulating L1 and S2. Reproduced with permission from Hunter and Yang [52].

### Other Postsurgical Pain

Thoracotomy surgery is an extremely common procedure associated with a high risk of severe chronic neuropathic postoperative pain [53]. Thoracotomy is performed for multiple conditions, and no data on the overall incidence of thoracotomy are available. However, lung or bronchus cancer is the most common reason for a thoracotomy, and the incidence of lung/bronchus cancer was estimated to be 234,030 cases in 2018 [54]. In recognition of the association of pain with thoracotomy, patients are typically provided with patient-controlled analgesia (PCA) pumps preemptively before even going into the operating room. Post-thoracotomy syndrome is defined as pain in the distribution of an intercostal nerve that persists after thoracotomy for at least three months after the procedure. In general, it is a burning and stabbing pain with dysesthesia, allodynia, hyper- and/or hypoesthesia and is seen as a neuropathic pain syndrome with damage to the intercostal nerve [55–57]. The published literature reports a wide range of occurrence, from 5% to 90%, likely due to the lack of consistent defining criteria, making the true incidence largely unknown or even underreported. Bayman and Brennan conducted a meta-analysis of prospective studies on chronic pain three and six months after thoracotomy and reported an incidence of 57% (95% confidence interval [CI] = 51–64%) and 47% (95% CI = 39–56%), respectively [58]. Reports of traditional SCS being used to successfully treat post-thoracotomy syndrome have been sparse [59]. Due to the extremely focal nature of this particular syndrome and the predictability of the involved nerves, DRG stimulation would appear to be a good option. To date, there has been only one preliminary case report of DRG stimulation being successfully used to treat post-thoracotomy syndrome in which the authors stimulated the DRG at the precise level of the injury (injury to T9 intercostal, T9 DRG lead placement) [60].

The foot is a known target for DRG stimulation, which has traditionally been difficult to capture with SCS. In the ACCURATE study [5], 79.3% of the causalgia subjects treated with DRG stimulation experienced ≥50% pain relief. Although the results were not reported by pain location, foot pain comprised 57% of the DRG subjects. Morgalla et al. reported long-term results of DRG therapy in 62 patients, 51 of whom went on to permanent implantation after a successful trial period. A total of 27 patients were implanted for postsurgical knee pain, six for postsurgical or post-traumatic foot pain, and 18 for other indications. For the subgroup of foot pain, it was not reported if they all met diagnostic criteria for CRPS. In the overall cohort, pain was reduced by more than 50% in 72% of patients at three years [38]. There appears to be potential to improve patient satisfaction and overall clinical outcomes with DRG stimulation to treat postsurgical foot pain, especially given the known drawbacks to dorsal column paresthesia-based stimulation [61]. Chronic foot pain after orthopedic or podiatric surgery is an area for future potential indication expansion of DRG stimulation.

## Discussion

Chronic postsurgical pain, often associated with a nerve injury, may not meet the traditional diagnostic criteria for CRPS I, although with a careful exam and documentation of neural injury, the diagnosis of causalgia (or CRPS II) can often be made. DRG stimulation appears effective for pain secondary to truncal nerve injuries not meeting all diagnostic criteria for CRPS. Increased awareness of postsurgical chronic pain syndromes has led to a search for the etiology and more treatment options. Recent research would suggest that many chronic postoperative pain states are related to neural injury [62]. Complex regional pain syndrome, types I and II, the primary indication for DRG stimulation in the United States, has proven difficult to define and classify [63]. A poorly understood constellation of signs and symptoms, the initial journey of uncertainty surrounding nomenclature saw terms such as “angiospastic syndrome,” “algodystrophy,” and “shoulder-hand syndrome” come and go [64]. More recently, seemingly interchangeable terms such as “reflex sympathetic dystrophy” and “Sudek’s atrophy” have given way to more uniform terminology. CRPS I is defined as without evidence of nerve injury and has replaced reflex sympathetic dystrophy, and CRPS II has replaced the term causalgia and is used in the setting of a known nerve injury [65]. The diagnosis of CRPS remains challenging. The current validated diagnostic guidelines were created in 2003; the Budapest Criteria for CRPS are summarized in Table 2 [66]. These criteria require the diagnosis of CRPS to be made with at least three of four symptoms and two of four signs in sensory, vasomotor, and sudomotor categories. However, signs involving things such as swelling, nail changes, and hair changes are difficult to elicit and interpret when dealing with anatomic areas outside of the extremities. In addition, CRPS II or causalgia requires a documented nerve injury. The truncal region, which is highly innervated and can be a site for neuropathic pain syndromes, does not routinely display these characteristics with nerve injury or nerve-related pain. The bias of the accepted diagnostic criteria toward the extremities has made the diagnosis of similarly behaving conditions on the trunk and proximal limbs extremely challenging [67]. As in the case of CPP, the anatomical regions affected may make clinical diagnosis using accepted criteria difficult [46].

**Table 2:** Budapest criteria for CRPS diagnosis

All of the following criteria must be met: Patient has continuing pain that is disproportionate to the inciting event Patient must have 1 sign in 2 or more categories below Patient must have 1 symptom in 3 or more categories below No other diagnosis can better explain the signs and symptoms	
Signs and Symptoms	
Sensory	Allodynia (pain from things that are not normally painful, e.g., light touch, temperature, deep somatic pressure, or joint movement) Hyperalgesia (heightened pain intensity from pinprick)
Vasomotor	Differences in skin temperature >1°C Differences in skin coloration on different sides of the body Skin color changes
Sudomotor/edema	Changes or asymmetry in swelling Changes of asymmetry in sweating
Motor/trophic	Decreased range of movement Motor symptoms (e.g., tremors, weakness, dystonia) Changes in hair, skin, nails (trophic)

CRPS = complex regional pain syndrome.

Clinical decision-making can be affected by this enigma. Level 1 evidence exists for the treatment of CRPS I and causalgia due to nerve injury (CRPS II) in the lower extremities with DRG stimulation [5]. However, clinical experience and emerging evidence show that effective treatment using DRG stimulation for neuropathic pain of the trunk and/or limbs and other postsurgical sites does not necessarily meet all the diagnostic criteria for CRPS type I or II [30,31,38]. A recent single-center case series of 62 patients with focal pain in multiple anatomies such as the knee, hand, foot, back, and leg provides the longest reported follow-up data to date. After a year, 82.5% (N = 33/40) of the implanted patients, and after three years, 72% of the implanted patients (N = 18/25), reported >50% reduction in pain [38].

In the ACCURATE study [5], in CRPS subjects, 82.5% of DRG subjects and 57.7% of SCS subjects experienced ≥50% pain relief (*P* < 0.006). For causalgia subjects, 79.3% of the DRG subjects and 53.3% of the SCS subjects experienced ≥50% pain relief (*P* < 0.014). Clearly DRG stimulation is effective in treating focal pain. At this time, neuropathic postsurgical pain of the groin, knee, and foot appear to be the conditions with the most clinical experience, backed by a limited but growing body of evidence. Additional potential indications include postamputation pain, postsurgical pelvic pain, and postsurgical pain after thoracotomy, hip replacement, and mastectomy. However, prospective studies lag behind real-world clinical experience and are needed to confirm these findings.

## Conclusions

In addition to CRPS types I and II, DRG stimulation continues to display strong responder rates and efficacy in the treatment of focal postsurgical neuropathic pain syndromes. Currently, NPP of the foot, groin, and knee appear to be the conditions with the most clinical experience, backed by a limited but growing body of evidence. Future prospective, statistically powered clinical studies will further clarify the role of DRG stimulation for postsurgical chronic pain after hernia surgery, hip and knee replacement, thoracotomy procedures, and phantom limb and stump pain. Many chronic postoperative pain states are focal and causalgic in nature, and the early data presented here suggest that DRG stimulation is a strong therapeutic option. However, prospective studies lag behind real-world clinical experience and are needed to confirm these observations.

## Notes

Funding sources: This study was funded by Abbott. Support was provided for travel to meetings to discuss the manuscript, and assistance with technical writing and editing.

Conflicts of interest: Dr. Antony is a Consultant for Abbott, has received research funding as a PI for Abbott and Nevro, has received research funding as a non-PI for Boston Scientific, and serves on the Product Advisory Board for Boston Scientific. Dr. Schultheis is a Consultant for Abbott and has received research funding as a PI for Abbott. Dr. Jolly is a Consultant for Abbott, Boston Scientific, and Nevro. Dr. Bates is a Consultant for Abbott, Nevro, and Nalu and serves on the Medical Advisory Board for Abbott. Dr. Hunter is a Consultant for Abbott, Saluda, and Nuvectra, is on the advisory board for Vertiflex, and has received research funding from Abbott, Saluda, Nuvectra, Biotronik, Mesoblast, Vivex, TissueGene, Vertiflex, and Neuros. Dr. Levy is a Consultant and has served on the Medical Advisory Board for Abbott, Mainstay Medical, Vertos, Nalu, Nuvectra, and Saluda.

Supplement sponsorship: This article appears as part of the supplement “Neuromodulation of the Spine and Nervous System” sponsored by Abbott.
